# Sow Nutrition, Uterine Contractions, and Placental Blood Flow during the Peri-Partum Period and Short-Term Effects on Offspring: A Review

**DOI:** 10.3390/ani13050910

**Published:** 2023-03-02

**Authors:** Moniek van den Bosch, Nicoline Soede, Bas Kemp, Henry van den Brand

**Affiliations:** 1 Adaptation Physiology Group, Wageningen University and Research, P.O. Box 338, 6700 AH Wageningen, The Netherlands; 2 Royal Agrifirm Group, Landgoedlaan 20, 7325 AW Apeldoorn, The Netherlands

**Keywords:** sow, parturition, placental blood flow, uterine contractions, energy requirements

## Abstract

**Simple Summary:**

A long birthing process is not only stressful for both the sow and her piglets, it also decreases the chances of survival of piglets during birth or in the first days of life. Oxygen supply from the mother to the fetus via placenta and umbilical cord is crucial. This oxygen supply may be impaired by successive uterine contractions, partly or completely blocking placental and/or umbilical blood flow. Providing the mother with the right amount of energy and other nutrients needed for the birthing process could reduce its duration and, as a consequence, increase peri-partum piglet survival. In addition, nutrients that enhance blood flow (and therefore oxygen flow) to piglets during birth may also impact piglet survival.

**Abstract:**

The birth process is a crucial event for piglet survival. Along with increasing litter sizes, not only has the duration of parturition increased, but placental blood flow per piglet has reduced and placental area per piglet has become smaller, making these piglets more susceptible for hypoxia. Diminishing the risk of piglet hypoxia by either reducing the total duration of parturition or increasing fetal oxygenation may reduce the incidence of stillbirth and early post-partum mortality. This review discusses options to do so by nutritionally supporting the sow in the final pre-partum period, after discussing the role of uterine contractions and placental blood flow. Providing sufficient energy seems to be a logical first step, but also other nutrients needed for uterine contractions, such as calcium, or enhancing uterine blood flow by using nitrate seem promising. These nutrient requirements may depend on litter size.

## 1. Introduction

The parturition process is challenging for both the sow and her piglets. For the sow, parturition is an energy demanding, stressful, and painful event [1]. For piglets, it is also a stressful event, and the odds of dying are highest during parturition and the first days of life [2]. The parturition process in sows has been studied mainly from a behavioral or endocrine point of view [3,4]. Only a few studies, however, have investigated peri-partum uterine contractions and placental or umbilical blood flow and the changing metabolic status and nutritional requirements of the sow during the peri-partum period [5]. Along with increases in litter size, the challenges to the perinatal piglet have increased, which are either related to in utero circumstances such as a decrease in uterine blood flow per piglet [6], a decrease in piglet [7,8,9] and placental size [2,10,11], or an increase in farrowing duration [12]. The survival rate for piglets mainly depends on fetal oxygenation, which in turn is related to farrowing duration [13,14], the duration and intensity of uterine contractions [15], and placental blood flow and therefore oxygen flow [16]. The maternal diet needs to provide the nutrients for uterine contractions and for sufficient placental blood flow, and its role has been investigated in recent studies [17,18,19,20,21,22]. This review focuses on possible interventions in the maternal diet that may facilitate the parturition process, affecting uterine contractions and/or placental blood flow in the perinatal period. 

## 2. Uterine Contractions 

**Figure 1:**
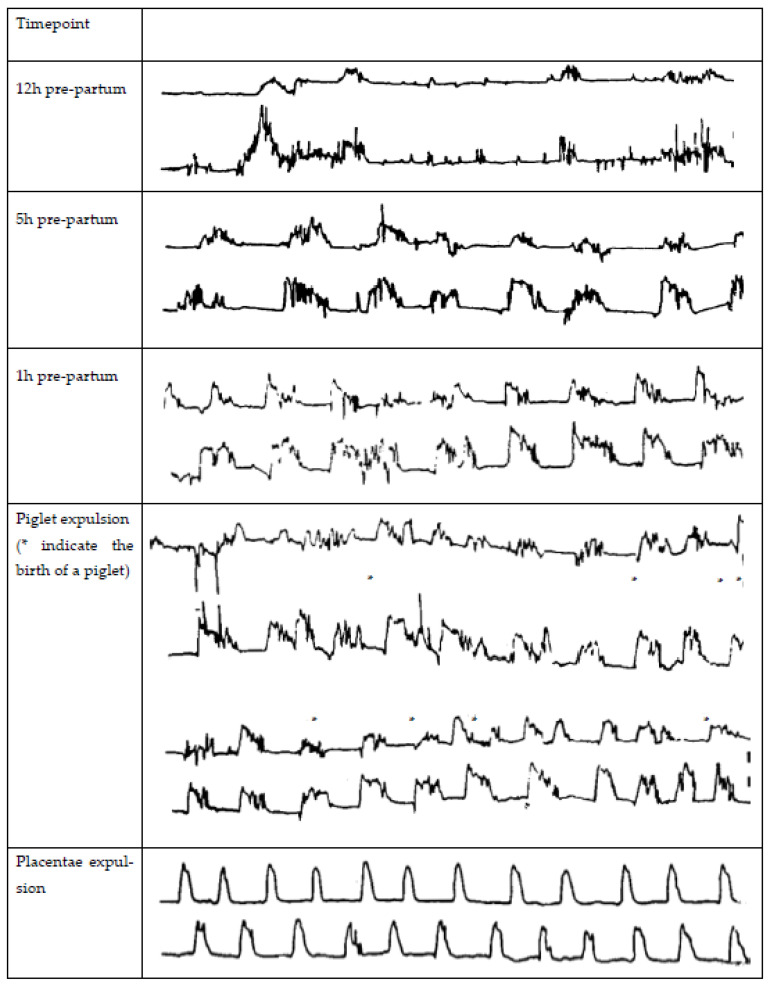
Myometrial contractions in the sow 12, 5, and 1 h pre-partum during spontaneous parturition (* indicate the birth of a piglet) and during the placentae expulsion, measured by two stain gauges implanted in each uterine horn as adapted from Maffeo et al. [15].

The parturition process in the sow can be divided into three stages: (1) increase in myometrial activity and dilation of the cervix (approximately 6–12 h), (2) expulsion of the piglets with the sow lying down and in abdominal straining (approximately 5–8 h), and (3) expulsion of the placenta (approximately 4 h, which may already start during stage 2) [12,23]. Several reviews discuss the complex endocrine changes in the peripartum period [24,25,26,27], so here, we only highlight the major changes. The increase in myometrial contractions in stage 1 results from a cascade of endocrine events. Fetal cortisol induces a release of endometrial PGF2α, which induces luteal regression and thereby results in a decline in progesterone. PGF2α also stimulates the release of relaxin by the corpora lutea, producing oxytocin and uterine smooth muscle contractions [28]. Exogenous prostaglandin injection to induce luteal regression and thereby induce parturition does not influence the parturition process itself. It is mainly a tool to optimize parturition management when given, at most, two days before parturition [29]. The placenta produces estrogens, and the changed progesterone/estrogen ratio increases the expression of oxytocin receptors on the myometrium, causing an increase in number as the ratio between progesterone and estrogen changes [27,30]. The changed progesterone/estrogen ratio stimulates myometrial contractions starting at 4–9 h before the expulsion of the first piglet. These contractions last for 2–3 min each and occur at regular intervals [31]. Contractions keep increasing in frequency and amplitude, and straining efforts of the sows start to appear the last few hours before expulsion of the first piglet [31]. As soon as the first fetus enters the cervix, stage 1 of parturition is considered to be completed [32]. Then, the Ferguson reflex is activated, releasing oxytocin from the pituitary. The increased oxytocin levels stimulate abdominal muscle straining to expel fetuses [33]. The frequency of uterine contractions is highest when piglets and the placentae are being expelled [34], but large variations occur among sows in frequency, duration, and amplitude and for an individual sow from one hour to the next of the expulsion phase [35]. During this phase, on average, uterine contractions last for 1–2 min and occur at a frequency of 18 per hour [34]. Maffeo et al. [15] gained insight into the frequency and amplitude of contractions during different timepoints of the parturition process using two implanted strain gauges (one in each horn) during spontaneous births. The frequency, amplitude, and duration of contractions 12, 5, and 1 h before the birth of the first piglet, during piglet expulsions, and during placenta expulsion are shown in Figure 1. 

Exogenous oxytocin injections can maintain and reinforce spontaneous parturition [36] by increasing the frequency of contractions 13-fold and their intensity 2-fold when compared to spontaneous contractions. This can result in a reduced duration of farrowing, but could also impair the normal physiology of contractions [37], which may result in a reduction in piglet vitality at birth and an increase in incidence of stillbirth [38]. This is likely due to damage of the umbilical cord [39] or a decrease in placental blood flow [40]. 

During the expulsion phase, tubo-cervical contractions move the fetuses towards the cervix. In addition, cervico-tubal contractions occur, which are likely meant to shorten the uterine horns and to prevent accumulation of fetuses at the caudal ends of the uterine horns [31] and/or to keep fetuses at a fixed place to keep the umbilical cord functional before expulsion [34]. Contractions are initiated at the two ends of the horns and convey (either as a tubo-cervical or cervico-tubal contraction) to the proximal end of the horns [34], but may rebound in the opposite direction when reaching the end of the horn [31]. Empty parts of the horn also contract [31]. Cervico-tubal contractions end when the horn is empty of piglets, indicating that the presence of piglets close to the cervix initiate these contractions [31]. It is estimated that four to five uterine contractions, with an average duration of 11.5 s and an intensity of 9.4 mm Hg, are needed to expel one fetus [38,41,42]. As soon as the horns are completely empty, contractions are only tubo-cervical and appear very frequent and regular for placentae expulsion [15,34]. It is unknown whether or not there is synchrony in the timing of contractions between the two horns, but this seems likely, since muscle fibers fuse at the common uterine body [43], and the birth order of fetuses from both uterine horns appears to happen fully at random from one horn or the other [34]. It is also unclear whether crowding of piglets occurs during contractions. It might be that crowding does occur when fetuses are stuck or when a stillborn piglet causes a delay in the birth process. The birth interval after which a stillborn piglet is born is approximately twice as long as that of a liveborn piglet (28 vs. 15 min) [14,44]. It is unknown whether the increase in birth interval is a cause or consequence of the increased birth interval [45].

## 3. Uterine Blood Flow

Most studies evaluating the duration of farrowing in sows only consider stage 2 of parturition, the time during which fetuses are expelled [12,14,17,22,46,47,48], as this stage determines the level of asphyxiation of piglets and can easily be observed. Asphyxiation mostly occurs due to strong uterine contractions combined with placental space limitation and/or reduced placental–uterine connection, which together reduce or obstruct placental blood flow [32]. As an initial response to reduced blood oxygen levels, fetal heartrate drops [49] and fetal movements increase, which in turn promote myometrial contractions, making a positive feedback system to reduce the duration of farrowing [32]. In fetuses with a prolonged inadequate oxygen supply, blood CO_2_ concentrations will rise, and hypoxia starts to occur. To reduce fetal oxygen consumption, not only will fetal limb and body movements reduce [50], but also fetal heartrate falls (bradycardia) [51,52] and metabolic rate reduces [52]. When fetal blood O_2_ concentration drops below a certain threshold level, adenosine triphosphate (ATP) production shifts to anaerobic glycolysis, and fetal lactate levels increase [51]. This anaerobe metabolism is faster than aerobe metabolism, but can only provide energy for a short period of time (up to 2 min) [53]. Lactate also lowers blood pH, which can affect functioning of the central nervous or cardiovascular system [54]. Lactate levels at birth have been related with chances of dying during lactation. For example, English and Wilkinson [55] showed that piglets that died pre-weaning had higher blood lactate concentrations at birth than survivors (383 vs. 303 μg lactate/mL blood; *p* < 0.01, respectively). Furthermore, Langendijk et al. [56] found a higher pre-weaning mortality when blood lactate concentrations in umbilical cord blood was increased (8.5% and 10.9% for 4.45–6.40 mmol/L and >6.40 mmol/L, respectively). Thus, the level of asphyxia at birth appears to be related to the chances for pre-weaning survival.

**Figure 2:**
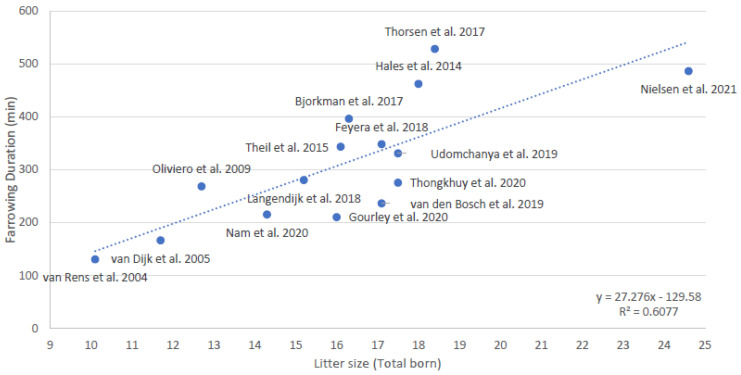
Relationship between average litter size and average duration of parturition (stage 2, expulsion of piglets) based on averages of 15 studies conducted over the last 18 years [12,14,17,46,47,57,58,59,60,61,62,63,64,65,66].

It is not known whether the number, duration, and amplitude of contractions, or the duration of stage 1 of parturition, is related to litter size. It is also not known whether the durations of stage 1 and 2 of parturition are related. It is known that the duration of stage 2 of parturition is related to litter size; it indeed takes more time to deliver more piglets [12]. Combining the data of 15 studies that measured the duration of stage 2 of parturition in the last 18 years [12,14,17,46,47,57,58,59,60,61,62,63,64,65,66] shows an estimated increase of 27 min in duration of stage 2 of parturition per extra piglet (Figure 2, averages per study). The deviation from the predicted line for farrowing duration based on litter size is sometimes quite large, which may be caused by differences in e.g., breed, housing, or management (i.e., use of birth assistance and exogenous hormones). 

Summarizing, the total duration of parturition (stage 1, 2, and 3), in which a sow experiences frequent and powerful uterine contractions, can take up to 24 h in the hyper-prolific sow [26]. Research on duration of parturition mainly focuses on phase 2 of parturition, i.e., the period during which the piglets are born, since this phase is the most easy to observe. It is unknown what the impact is of phase 1 on the sow, her piglets, and how related phase 1 and 2 of parturition are with each other. Most of the studies investigating the intensity, number, and duration of uterine contractions for the different phases of parturition in the sow were done three to four decades ago [15,31,34]. It is unknown whether the intensity, number, and duration of uterine contractions relate to the current litter sizes and other aspects of the current highly prolific sow. 

## 4. Placental and Umbilical Cord Functionality

The placenta is responsible for nutrient and oxygen exchange between the sow and her fetuses. The fetus has a diffuse placenta in which many closely spaced chorionic villi are distributed over the entire outer surface of the chorion [32], which ensures transport and diffusion of nutrients from the maternal to the fetal blood. Additionally, specific structures called areolae on the placenta absorb the products secreted by the endometrial glands (e.g., growth hormones, hormones, transport proteins lymphokines, cytokines) [32]. The surface area of the chorio-allantoic membrane mainly increases in size between day 35 to 70 of gestation, with little change between day 70 to 100 of gestation [10]. Vascularization of the allantoic membrane starts at approximately day 15 post-insemination, i.e., 2 days after contact between the trophoblast and maternal epithelium [67], and increases until mid-gestation, after which vascularity remain relatively constant [68,69]. By that time, blood vessels occupy about 3–4% of the chorio-allantoic membrane, but with large variation among individual fetuses, among litters, and between breeds [68,69]. Blood capillaries from the chorionic villi merge and eventually form larger vessels that enter the umbilical cord [32]. In addition to vascularization, nutrient supply to the fetus is also affected by uterine blood flow, which increases as gestation progresses [6]. Although it seems likely, it is not known, whether vascularization of the placenta and placental blood flow are related. Blood flow [70] and placental area [2] per piglet both seem negatively correlated with litter size, which likely explains why average piglet birth weight decreases as litter size increases [7,9,71]. No studies were found showing a clear relationship between litter size and placental vascularization. Wilson et al. [69] found differences between breeds in placental vascularization at the fetal–maternal interface. Vascular density was higher in Meishan placentas compared to Yorkshire placentas, although placental size was larger in Yorkshire sows. Whether placental blood flow differs between breeds has not been evaluated. 

Placental characteristics and the incidence of pre-weaning mortality appear to be related, although these relationships might be confounded with piglet birth weight. Both placental surface (−20.4%) and placental weight (−14.8%) were lower in piglets that died before weaning compared to surviving conspecifics, which was most likely caused by a lower birth weight of piglets that died before weaning [2]. Baxter et al. [72] found no difference in vascularization score of placentas of piglets that survived or died before weaning.

The umbilical cord connects the fetus to the placenta, and it contains one vein that carries oxygen and nutrient-rich blood to the fetus and two smaller arteries that transport deoxygenated blood from the fetus back to the placenta [73]. These vessels are surrounded by Wharton’s jelly, a gelatinous connective tissue consisting mainly of hyaluronic acid, in which collagenous and reticular fibers form a loose meshwork [74]. An intact and functional umbilical cord is of crucial importance for fetal oxygen and nutrient supply. Umbilical cord length of piglets was found to be 35 cm on average (ranging between 17 to 50 cm) and was positively correlated with piglet weight [9]. Umbilical cord length is not correlated to the position of a piglet in the uterus, but its elasticity (up to 37.5% of its length) allows it to stretch as a piglet is transported through the uterine horn at parturition, making it possible for piglets at the end of the uterus to be born with intact umbilical cords. The tension required to break an umbilical cord varies from 545 to 2000 g [75,76]. The percentage of piglets born with a broken umbilical cord lies between 21 to 71% [9,56], and Rootwelt et al. [9] showed that broken umbilical cords occur most in the second and last third of piglets born (2.3 times more often compared to the first third of piglets born). When or where an umbilical cord breaks has, to our knowledge, not been studied in pigs. It can be hypothesized that the umbilical cord breaks at a weak spot or occurs randomly over the full length of the umbilical cord, potentially caused by a weak spot in the Wharton’s jelly or the first place where umbilical cord blood flow has stopped. It is also unclear which placental or other sow and/or piglet characteristics might be related with umbilical cord length, thickness, strength, or breaking point. It seems likely that larger piglets, which have a larger placenta [2,9], also have a thicker umbilical cord that may also be less prone to breaking. Curtis et al. [77] suggested that stillborn piglets (that weighed less than live-born litter mates) have a smaller umbilical cord that is more likely to break, suggesting a relationship with piglet birth weight and umbilical cord thickness/strength. However, Langendijk and Plush [49] found a similar weight distribution in live and stillborn piglets, suggesting that the hypothesis of Curtis et al. [77] might not be true. Piglets born alive but with a broken umbilical cord (as observed at the moment of birth) showed a lower vitality score and had an higher risk for post-partum death compared to piglets born with an intact umbilical cord [2]. A recent review estimated the association between incidence of stillbirth and a broken umbilical cord before expulsion to be 50% or more [49]. In addition, even when the cord does not break, extensive stretching might lead to vasoconstriction and limited blood flow, increasing the risk for stillbirth [39]. 

In summary, in larger litters, placental blood flow per piglet is reduced [70] and placental area per piglet is smaller [2], which likely explains why average piglet birth weight is lower [7,9,71] and partially explains why incidence of pre-weaning mortality increases. An intact and functional umbilical cord is key for fetal oxygen and nutrient supply and therefore survival. Studies on how, where, or when an umbilical cord breaks and which sow and/or piglet characteristics are related to its breaking are limited. A better understanding of the complex interactions between placental/umbilical cord blood flow, contractions, breaking of the umbilical cord, and other characteristics of the modern sow might provide insights in how perinatal piglet losses can be reduced. In conclusion, placental and/or umbilical cord blood flow might be under pressure in large litters, which might be related to stillbirth and pre-weaning mortality. 

## 5. The Potential of Maternal Nutrition to Reduce Farrowing Duration

**Figure 3:**
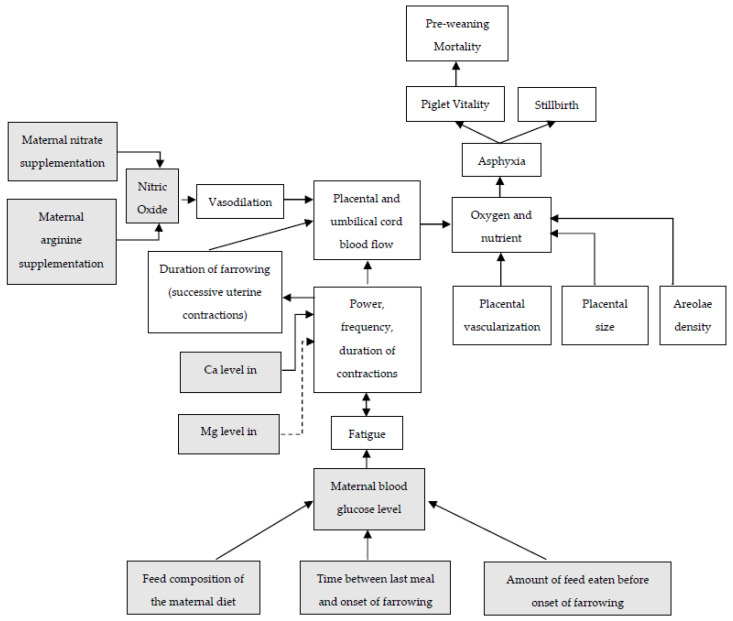
Relationships between farrowing and placental characteristics on piglet vitality, incidence of stillbirth and pre-weaning mortality, and potential roles of maternal nutrients on these relationships. Dotted lines indicate a negative effect. Grey boxes will be discussed in current paragraph.

Relationships between placental characteristics, uterine contractions, placental and umbilical cord blood flow, and piglet losses are summarized in Figure 3. Additionally, in this figure, the potential effects of maternal nutrients on these events are included. Providing the right nutrients to the sow and her fetuses may not only enhance placental development and fetal growth but could also affect uterine blood flow in the perinatal period and/or affect the duration of farrowing. Studies evaluating nutritional solutions aiming to reduce farrowing duration by enhancing uterine contractions or affect placental characteristics and therefore affecting piglet losses during or shortly after parturition will be discussed in the next paragraph.

Nutritional interventions in the perinatal period aiming to decrease stillbirth and to increase piglet vitality right after birth should stimulate uterine contractions (frequency or intensity), increase placental nutrient and/or oxygen supply to the fetus, and/or provide the sow with the energy to prevent fatigue. To prevent constipation [78] and metritis, mastitis and agalactia (MMA) [79] feed allowance in some European countries is lowered to 2.0–3.0 kg/sow/day, beginning 2–3 days before the expected farrowing date. It can be questioned whether this lower energy and nutrient intake and the type of nutrients supplied sufficiently facilitates energy and nutrient requirements during parturition. Consequently, feeding strategies in the perinatal period might need to be reconsidered. 

### 5.1. Energy

The total duration of farrowing (stage 1, 2, and 3) can take up to 24 h in the hyper-prolific sow [26] and is positively related to litter size [80]. When we expect our sows to give birth to larger litters, we should provide them with the right nutrients to perform this activity. Focus on energy requirements seems to be a logical first step, since farrowing is likely a highly energy-demanding activity [61,81]. It seems likely that modern sows do experience a limitation in available energy around farrowing. Van Kempen et al. [82] suggested that sow exhaustion during farrowing caused by energy depletion could impair the number and intensity of uterine contractions, thereby increasing the duration of farrowing and consequently increasing stillbirth rate. That sow exhaustion occurs was also suggested by Mosnier et al. [5], who found a higher sow plasma lactate concentration at day 1 post-partum (approximately 1.4 mmol/L) compared to day 4 (approximately 0.9 mmol/L). The higher concentration of lactate in sow blood is likely due to increased metabolic activity and uterine contractions of sows during farrowing (also seen by an increase in body temperature [83]). A recent study, in which lactate levels were determined more frequently around parturition (every 6 h pre-farrowing and every 3 h post-partum), showed that sow blood lactate levels were indeed increased during parturition, but were already increased at 9 and 3 h before the expulsion of the first piglet [65], which is likely related to higher activity during nest-building behavior and by increased uterine contractions during phase 1 of parturition. Three hours after farrowing, lactate levels started to decrease again [65], indicating that sows shifted back to their aerobe metabolism. 

**Figure 4:**
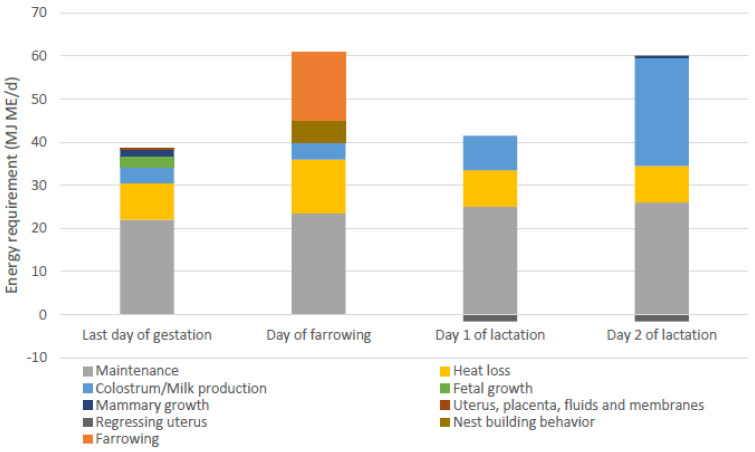
Energy requirements of sows on the last day of gestation, the day of farrowing, and day 1 and 2 of lactation, adapted from [59], including estimated energy requirements for additional heat loss, nest building behaviour and farrowing [81]. ME = metabolizable energy.

The energy requirement for the farrowing process is expected to be comparable to moderate to heavy exercise [61]. Recent estimates of energy requirements estimated during the transition period (10 days pre-farrowing to 10 days post-farrowing) included maintenance, heat loss, mammary growth, fetal growth, and colostrum/milk production, but not requirements for the farrowing process itself, resulting in the lowest estimated energy requirements at the day of farrowing [59]. Other recent evaluations of amino acid and energy requirements also did not include parturition requirements [84]. Feyera et al. [81] were the first to give an estimate of energy requirements of farrowing, which was based on an evaluation of different feed amounts and therefore daily energy intake around farrowing, aiming for the shortest farrowing duration and lowest number of interventions during farrowing. The estimated energy requirement for farrowing was 16 MJ ME (approximately 30% of the total energy requirements on the day of farrowing). In Figure 4, the calculated energy requirements of sows provided by Theil et al. and Feyera et al. are combined for the last day of gestation, the day of farrowing, and day 1 and 2 of lactation. These estimates included energy requirements for maintenance purposes, heat loss due to reproduction costs and diet induced thermogenesis [85,86], colostrum/milk production, fetal growth, mammary growth, growth of uterine tissue, nest-building behavior, and energy requirements for farrowing.

In addition to the study by Feyera et al. [81], Che et al. [87] evaluated effects of energy intake on the day of farrowing on farrowing duration. Strategies of how the energy intake was increased differed between these two studies. Che et al. [87] increased energy level of the diet by increasing fat levels (soybean oil) and increasing daily feed supply with 0.2 kg/sow/day (from day 90 of gestation until farrowing), while Feyera et al. [76] increased feeding levels (from 1.8 to 5.0 kg/sow/day from day 108 of gestation until 24 h after farrowing). Both studies also differed in average litter size and average farrowing duration. Because farrowing duration increases with increasing litter size (Figure 2), it can be assumed that energy requirements for farrowing also increase with litter size. For estimating the energy requirements per piglet born or per 60 min of farrowing, it was assumed that the energy requirements were met when farrowing duration was shortest. For the treatments with the shortest farrowing duration, number of piglets born and average farrowing durations are shown in Table 1. Calculations on average energy requirement per piglet and per 60 min for both studies turned out to be quite close. Calculations suggest that optimal daily energy intake on the day of farrowing depends on the average litter (2.44 MJ ME/piglet born) and/or farrowing duration (8.66 MJ ME/hour of farrowing duration). Since only two studies were available, the findings should be confirmed in additional experiments. 

### 5.2. Glucose as a Source of Energy during Farrowing

ATP (adenosine triphosphate), derived primarily from glucose by glucogenesis, is the main energy source for uterine contractions [88]. Blood glucose levels rise during farrowing, which can be explained by the increased glucolysis under the influence of adrenalin and cortisol [19,89]. Sow blood glucose levels originate from carbohydrates in the diet and/or glycogen reserves. A negative correlation was found between sow arterial glucose level, measured 1 h after the birth of the first piglet and farrowing duration, suggesting that a low energy status of the sow indeed increased farrowing duration. Feyera et al. [17] showed that farrowing duration linearly increased with time when the last meal was more than 3.13 ± 0.34 h before the onset of farrowing (defined as the birth of the first piglet). Theil et al. [37] showed that sows lack glucogenic energy on the day of farrowing and start using the glycerol part of triglicerides as an energy source (rather than nonesterified fatty acids in a normal catabolic state), making triglycerides and glucose the only two energy sources for the uterus during farrowing [37,90]. 

Although duration of farrowing has been related to the energy status of the sow, other dietary factors (e.g., type of energy, mineral levels, other supplements) might play a role as well. These factors will be discussed below.

### 5.3. Other Carbohydrates

The role of dietary fibers in sow nutrition around farrowing are mostly studied in relation to the prevention of constipation and therefore easy passage of piglets through the birth canal [18,58]. Effects of dietary fibers on the duration of farrowing have been reviewed extensively [25] and will thus not be discussed here. No information is available on possible effects of dietary fibers on uterine contractions. However, dietary fibers can be a source of energy from the gastrointestinal tract up to several hours after a meal [91]. The type of carbohydrates consumed appears to be an important factor for exercise performance in athletes [92]. The glycemic index (GI) of carbohydrates is a tool to predict blood glucose, insulin, and therefore energy supply of diets [93] High GI foods (e.g., sugars and starches) provide a high and relatively short peak in blood glucose. Low GI feed ingredients (e.g., pectins) could provide lower but longer levels of blood glucose. Combining different types of carbohydrates, providing fast-, medium-, and slow-release glucose, might increase the period after feeding in which sufficient glucose is available to supply the energy needed for the farrowing process. 

It can be concluded that a better understanding of perinatal energy requirements (and the composition of these energy sources) is needed to optimize the farrowing process and consequently reduce piglet losses.

### 5.4. Calcium and Magnesium

Calcium is an essential mineral for muscle contractions [94,95] and therefore also essential for myometrial contractions during farrowing (Figure 3). Studies evaluating calcium requirements in the peri-partum period are limited. Geisenhauser et al. [96] evaluated effects of a single-dose calcium supplementation on top of feed (400 mmol Ca, source calcium lactate) on the day of farrowing and found a significant reduction (−34% on sow level) in the incidence of dystocia (defined as birth interval > 60 min) and decreased time for placenta expulsion (4–19 min faster). Le Cozler et al. [19] evaluated plasma calcium levels in gilts before, during, and after farrowing and observed no change in plasma calcium levels during parturition (measuring 2 h before the birth of the first piglet to 7 h after). This is in contrast to a recent study of Nielsen et al. [65], who also evaluated plasma calcium profiles around parturition (measuring 33 h before the birth of the first piglet until 24 h after farrowing) and found a drop in calcium levels 9 and 3 h before the expulsion of the first piglet, but no changes in sow blood calcium levels during the first 24 h post-farrowing. These ambiguous results in perinatal plasma calcium profiles might be related with differences in litter size (12.2 vs. 24.6 total born for Le Cozler et al. [97] and Nielsen et al. [65], respectively) and/or farrowing duration (175 vs. 486 min for Le Cozler et al. [97] and Nielsen et al. [65], respectively), suggesting that hyper-prolific sows might have higher calcium requirements in the perinatal period. The benefits of calcium supplementation to sows before farrowing on incidence of stillbirth and piglet vitality remain unclear but might be related to the plane of feeding in this period and the calcium source and concentration in the diet. 

Magnesium promotes the relaxation of smooth muscle cells and inhibits contractions of the uterine myometrium. Magnesium sulphate is used in human medicine to prevent pre-term labor and pre-term birth [98], which suggests that magnesium supplementation before farrowing might have a negative effect on myometrial contractions. However, Le Cozler et al. [19] observed a drop in magnesium levels in sow blood 1 h after the first piglet was born, which was likely due to the role of magnesium in dephosphorylation of ATP to provide energy for muscle contractions, as ATP must be bound to a magnesium ion to be biologically active. The synergistic and antagonistic role of magnesium with calcium might explain why calcium levels were constant and magnesium levels dropped during parturition [97]. In pig husbandry, magnesium is used in sow diets as an effective laxative to prevent constipation [99]. However, as with calcium, research on magnesium supplementation for sows in the perinatal period is limited. Plush et al. [99] showed an increase in stillbirth incidence (+0.3 stillborn piglets/litter, *p* = 0.01) when sows were supplemented with magnesium sulphate (2.85 kg/mton feed, receiving 2.5 kg of feed/sow/day) from 5 days pre-farrowing until 3 days post-farrowing. It can be speculated that magnesium induced relaxation of the myometrium, which consequently increased the duration of farrowing, but this was not evaluated.

Vitamin D is essential for intestinal calcium absorption, plays a central role in calcium homeostasis, and directly impacts muscle contractions [100,101]. Vitamin D is usually added to sow diets in the form of cholecalciferol (vitamin D_3_), which is transported to the liver and hydroxylated to 25-hydroxycholecalciferol [25(OH)D_3_], or by directly feeding the 25(OH)D_3_ [102]_._ Although requirements for sows are known for gestation and lactation [103], vitamin D requirements specifically during parturition are not. Some studies evaluated maternal vitamin D supplementation on offspring status in muscle fibers and therefore lean development and growth performance in later life [104,105,106,107]. However, we found no studies that evaluated maternal vitamin D supplementation and the effects on parturition characteristics and piglet losses. 

It can be concluded that although both calcium and magnesium play an important role in myometrial contractions, research on supplementing sows with one or both of these minerals in the perinatal period is very limited. Consequently, calcium and magnesium requirements of the sow in the perinatal period and potential factors influencing these requirements (e.g., litter size) are currently unknown.

### 5.5. Vasoactive Components

Dietary arginine (as recently reviewed by [108,109]) and nitrate supplementation [61,110] to the sow both aim to influence placental vascularization and/or placental–fetal blood flow. Although converted differently, both arginine (oxidized in a reaction catalyzed by the NO synthase family [111]) and nitrate (non-enzymatically converted via the NO_3_-NO_2_-NO pathway [112]) are precursors for nitric oxide (NO). NO is an endothelium-derived relaxing factor, causing vascular vasodilation [113,114], which plays an important role in regulating placental–fetal blood flow and consequently nutrient and oxygen transfer from mother to fetuses [37,115]. This higher blood, and therefore nutrient and oxygen, flow may lead to an increased piglet birth weight and/or oxygenation during parturition, which is hypothesized to lead to a lower incidence of stillbirth, increased vitality, and therefore a decreased incidence of pre-weaning mortality. Arginine is mostly supplemented in the first stage of gestation to increase placental angiogenesis [116], with several studies showing a beneficial effect on embryo survival, fetal development, placental weight, piglet weight, and number born alive (as reviewed by [108,117]). Fewer studies have used arginine supplementation up to or close to the moment of parturition. Neither placental weight (when supplementing 1% of L-arginine from day 22 until day 114 of gestation [118]) nor piglet birth weight and stillbirth rate (when supplementing 25.5 g/d from day 77 of gestation until term [119]) were affected in these studies. Van den Bosch et al. [61,110] evaluated effects of dietary nitrate supplementation starting 7 days before farrowing and found a linear dosage effect on piglet vitality (by scoring individual piglet vitality [72]) and piglet birth weights and a tendency for a lower pre-weaning mortality rate, which may have been driven by an increased placenta size and/or vasodilation. The use of NO precursors to enhance either placental vascularization and/or blood flow may benefit piglet vitality and survival.

## 6. Conclusions

Parturition is not only a stressful, painful, and energy-demanding event for sows, but it also affects the perinatal survival of her offspring. Along with increases in litter size, farrowing duration has increased and uterine blood flow per piglet, placental development, and piglet weight have decreased, which has increased the challenges to the perinatal piglet. Potential maternal nutritional factors that stimulate uterine contractions and/or increase uterine blood flow (by providing adequate energy and/or minerals or by the use of NO precursors) may reduce the duration of parturition and/or increase perinatal piglet survival. However, knowledge on the exact nutritional requirements before, during, and after parturition and the impact that meeting these requirements may have on piglet characteristics and perinatal survival is limited. Current feeding strategies in the perinatal period might not support the modern hyper-prolific sow adequately in energy and other nutritional requirements. 

## Figures and Tables

**Table 1:** Calculation on estimated energy requirements on the day of farrowing per piglet and per 60 min of farrowing based on Che et al. [87] and Feyera et al. [81].

Study	Che et al. [87]	Feyera et al. [81]	Average
Average litter size	14.8	20.4	
Average farrowing duration (min)	238.4	359.8	
Optimal daily energy intake determined based on the shortest duration of farrowing (MJ ME/sow/day)	33.8	53.0	
Energy requirement on the day of farrowing per piglet (MJ ME)	2.28	2.60	2.44
Energy requirement on the day of farrowing per 60 min of farrowing (MJ ME)	8.49	8.83	8.66

## Data Availability

The data presented in this review is available on request from the corresponding author.
